# Hashtag Global Surgery: The Role of Social Media in Advancing the Field of Global Surgery

**DOI:** 10.7759/cureus.8468

**Published:** 2020-06-06

**Authors:** Dominique Vervoort, Jessica G Luc

**Affiliations:** 1 Department of Health Policy and Management, Johns Hopkins Bloomberg School of Public Health, Baltimore, USA; 2 Division of Cardiovascular Surgery, University of British Columbia, Vancouver, CAN

**Keywords:** global surgery, social media, global health

## Abstract

Introduction: Surgery is increasingly recognized as an indispensable part of healthcare, but lack of awareness about its cost-effectiveness and cross-cutting impact remain. Social media has become an important resource for healthcare professionals in a variety of settings due to its instant global reach in a non-discriminatory and low-threshold manner. In 2010, #globalsurgery was first used on Twitter to spread awareness, foster international collaborations, and raise voices of advocates around the world. Here, we examine the role of social media in the field of global surgery.

Methods: The use of #globalsurgery on Twitter was analyzed through Tweetreach from July 31 to December 31, 2018. Additional analysis of hashtags in Spanish, Japanese, Malay, and Portuguese was done to determine the number of tweets, retweets, impressions, and users using #globalsurgery or translated hashtags. Sentiment analysis was performed to determine the affective state of tweets.

Results: A total of 4,519 tweets and 15,861 retweets were posted by 4,449 different contributors. Tweets totalled 58,733,406 potential direct impressions and 46,560,293 potential amplified impressions, with potential reach of 11,272,014. English was the major language (99.47%), followed by Spanish (0.49%) and Japanese (0.04%). Portuguese and Malay hashtags were not used during the study period.

Conclusion: #globalsurgery provides an innovative way to overcome barriers and strengthen collaboration among advocates, and more effectively raise awareness about global surgery.

## Introduction

It is estimated that five billion people worldwide lack access to safe surgical care when needed, responsible for over 17 million preventable deaths per year and one-third of the global burden of disease [1]. Although cost-effective on an individual and macroeconomic level, misperceptions and lack of awareness about the cost-effectiveness, feasibility, and cross-cutting impact of scaling up surgical care remain. Global surgery, as defined by Dare et al., is an area for study, research, practice, and advocacy that places priority on improving health outcomes and achieving health equity for all people worldwide who are affected by surgical conditions or have a need for surgical care [2].

Over 40% of adults in the world own a smartphone and most of their time is spent using the internet and social media [3]. Today, there are 2.62 billion social media users, including 2.23 billion Facebook users, 330 million Twitter users, and 260 million LinkedIn accounts, leaving the potential reach beyond imagination [4]. Currently, social media is widely used in healthcare for conference tweeting, journal clubs, coordination of research collaborative groups, dissemination of new research from peer-reviewed journals, medical education, and social and medicopolitical campaigns, among other pursuits [5-12]. It allows for real-time dialogue and collaboration across the world on any topic at each individual’s convenience in an asynchronous matter. Accordingly, social media provides a powerful tool to increase the body of knowledge for both patient care and safety, and broader areas, including research, diplomacy, and advocacy skills for health professionals, policymakers, and other health advocates.

Twitter is a free social network, commonly using hashtags (words following the symbol #) to denote keywords and allow the grouping of tweets according to common topics [13]. Its asynchronous nature allows for research, knowledge, and conference insights to be accessible across borders and timezones, with the ability to multiply the impact of scholarship through additional impressions. Twitter is the most frequently used smartphone application by the surgical community, although this varies between specialties [14].

In 2010, the #globalsurgery hashtag was first used, inadvertently starting a campaign to (1) spread awareness about the topic of global surgery amongst medical professionals, policymakers, funders, and the wider public; (2) foster international collaboration amongst surgical specialists through the creation of a virtual international community with a shared interest in advancing surgical care around the world; and (3) raise and echo the voices of global surgery champions, advocates, and patients. Following this, PubMed publications explicitly discussing “global surgery” increased from three in 2010 to 153 in 2018, whereas institutions in the United States including global surgery electives in their surgical residency programs surged significantly in the past decade [15].

Here, we perform a feasibility study to describe the use and impact of the #globalsurgery campaign to guide future efforts of strengthening and optimizing social media engagement in the growing field of global surgery.

## Materials and methods

This study aims to determine the use and impact of an online community of global surgery using Twitter. The use of the #globalsurgery hashtag was prospectively analyzed through Tweetreach for a period of five months from July 31, 2018 to December 31, 2018. Tweetreach is an online tool integrated into Twitter and assimilates data on hashtags upon request [16]. The use of translated hashtags in four of the top five non-English languages on Twitter (Spanish, Japanese, Malay, and Portuguese) was also determined during this period [17]. Analysis of tweets in English, Spanish, Japanese, Malay, and Portuguese was done to determine the number of original tweets, retweets, impressions, and users using #globalsurgery or any of the four translated hashtags (＃グローバルサージェリー, #CirugiaGlobal, #PembedahanGlobal, #CirurgiaGlobal). The Arabic hashtags were excluded from the analysis because of the variety of Arabic dialects in written communication.

Emotion-based sentiment analysis, or opinion mining, was applied to the data on hashtag use. Sentiment analysis determines the affective state or judgement of the user through the phrasing used in the whole message in which the hashtag is used through natural language processing through Tweetreach as previously described [18].

A dedicated #globalsurgery tweetchat was held on February 6, 2019, which followed shortly after the selected study period. TweetChat analytics was measured using Symplur (Symplur LLC, Los Angeles, CA, USA). Data variables included the number of participants in the TweetChat, total tweets, and impressions. Followerwonk (Marc Mimms, Spokane Valley, WA, USA) followerwonk.com and Twitter analytics were used to analyze various global surgery Twitter account activity and followers. Trendsmap (Trendsmap, Melbourne, Australia) was used to map the geographic distribution of original tweets.

## Results

**Figure 1:**
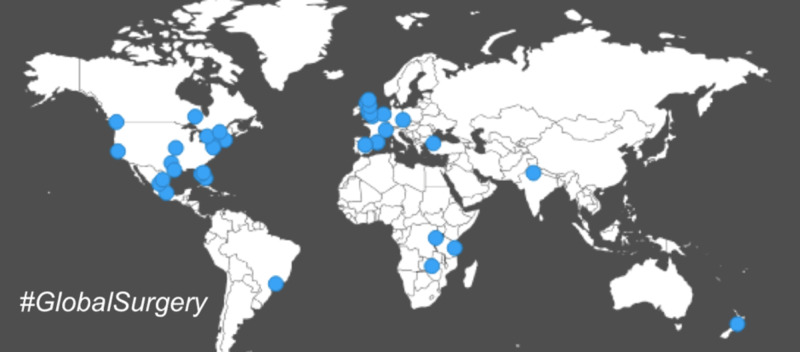
Geographic location of origin of tweets containing #globalsurgery. The blue dots depict the location associated with Twitter accounts using #globalsurgery.

Overall, 4,519 tweets (4,004 original tweets and 515 tweet replies) and 15,861 retweets were posted between July 31, 2018 and December 31, 2018 by a total of 4,449 different contributors. Original tweets had a total of 58,733,406 potential direct impressions (total number of views generated by original tweets). Retweets had a total of 46,560,293 potential amplified impressions (total number of views generated through retweets). Altogether, tweets containing the #GlobalSurgery hashtag had a potential reach of 11,272,014 (the unique potential audience that viewed #GlobalSurgery tweets) with global representation (Figure 1).

The analysis of hashtags in the major languages is summarized in Table 1. English was observed as the overall major language during the five-month period (99.47%). Spanish (0.49%) and Japanese (0.04%) were only used sporadically. No tweets containing the Portuguese and Malay hashtags were found.

**Table 1:** Analysis of global surgery hashtags. Analysis of global surgery hashtags in English and the top five non-English languages on Twitter between July 31, 2018 and December 31, 2018. *Users that used the translated global surgery hashtags also used #GlobalSurgery. N.A. = not applicable.

Language	Hashtag	Total number of tweets	Total number of retweets	Number of different users	Total number of potential impressions
English	#GlobalSurgery	4,495	15,798	4,449	58,714,799
Japanese	＃グローバルサージェリー	2	6	2	1,236
Spanish	#CirugiaGlobal	22	57	10	17,371
Malay	#PembedahanGlobal	0	0	0	0
Portuguese	#CirurgiaGlobal	0	0	0	0
Total	N.A.	4,519	15,861	4,449^*^	58,733,406

The top 10 contributors defined by direct impressions (@WebMD, @juliomayol, @WorldBank, @Lawrence, @HarvardPGSSC, @JohnMeara, @GlobalSurg, @theG4Alliance, @CelestinoGutirr, @DVervoort94) were collectively responsible for a contribution of 2,131 tweets (47.4% of total) and 21,942,944 potential direct impressions (37.37% of total) (Table 2). These numbers, in addition to the respective numbers of followers, act as indicators for the social reach of these individuals and organizations. In addition, their engagement (2,493 retweets) and amplification (28,173,207 potential amplification impressions) made up 15.78% and 60.51% of the total number of retweets and potential amplification impressions, reflecting the influence of these accounts.

**Table 2:** Top 10 #globalsurgery contributors (defined by direct impressions) between July 31, 2018 and December 31, 2018. Analysis of contribution, engagement, and amplification by the top 10 #globalsurgery contributors (defined by direct impressions) between July 31, 2018 and December 31, 2018.

Contributor	Followers	Tweets	Potential direct impressions	Retweets	Potential amplification impressions
@WebMD	3,060,209	1	3,060,209	9	3,067,559
@juliomayol	24,935	130	2,993,562	33	3,207,720
@WorldBank	2,860,905	1	2,860,905	50	2,913,491
@Lawrence	2,479,544	1	2,479,544	0	2,479,544
@HarvardPGSSC	6,328	384	2,263,819	1,043	5,005,339
@JohnMeara	5,754	412	2,248,141	183	3,082,991
@GlobalSurg	7,347	312	2,192,028	153	2,294,496
@theG4Alliance	5,284	299	1,534,422	203	2,085,727
@CelestinoGutirr	7,655	171	1,257,961	0	1,257,961
@DVervoort94	2,675	420	1,052,353	819	2,778,442
Total		2,131	21,942,944	2,493	28,173,207

Sentiment analysis for the total set of tweets, including the relevant hashtags within the five-month period, detected an overall neutral sentiment score of 62 (out of 100). Positive messages had a 44.1% share of the total set, compared to a large proportion of neutral messages with a 43.8% share and only a small proportion of negative messages with a 12.1% share, respectively. This indicates that most messaging makes use of positive (e.g., lives saved) and neutral phrasing (e.g., number of surgical specialists), with only limited relative use of negative writing (e.g., over 17 million deaths each year).

**Figure 2:**
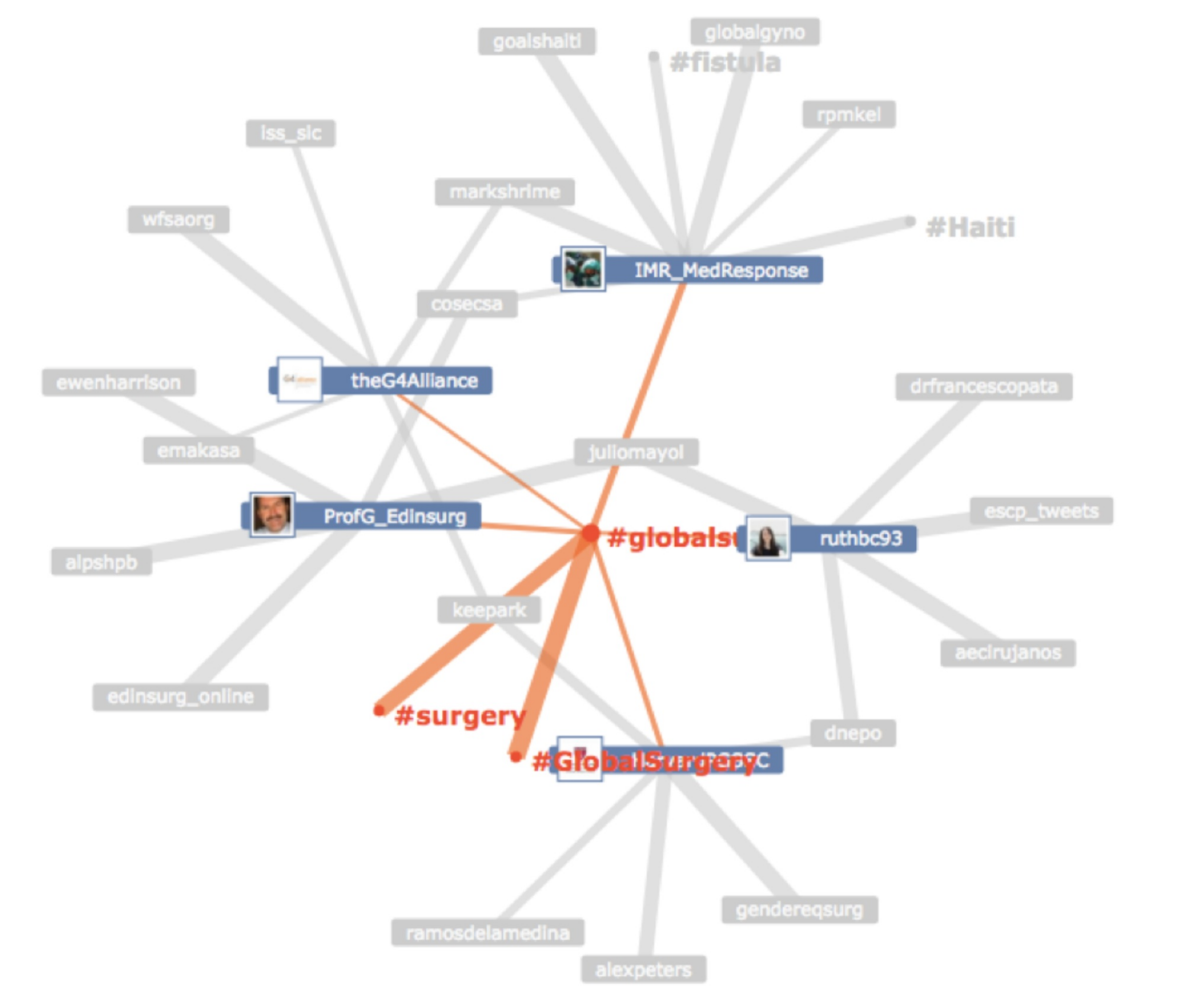
#GlobalSurgery node-edge diagram. Node-edge diagram illustrating communication amongst participants and other related hashtags using the #globalsurgery hashtag. Participants (blue) and hashtags (orange) in colour have higher activity with #globalsurgery hashtag than do the ones in grey. The arrows and the size of the arrows connecting users correlated with the activity of communication.

**Figure 3:**
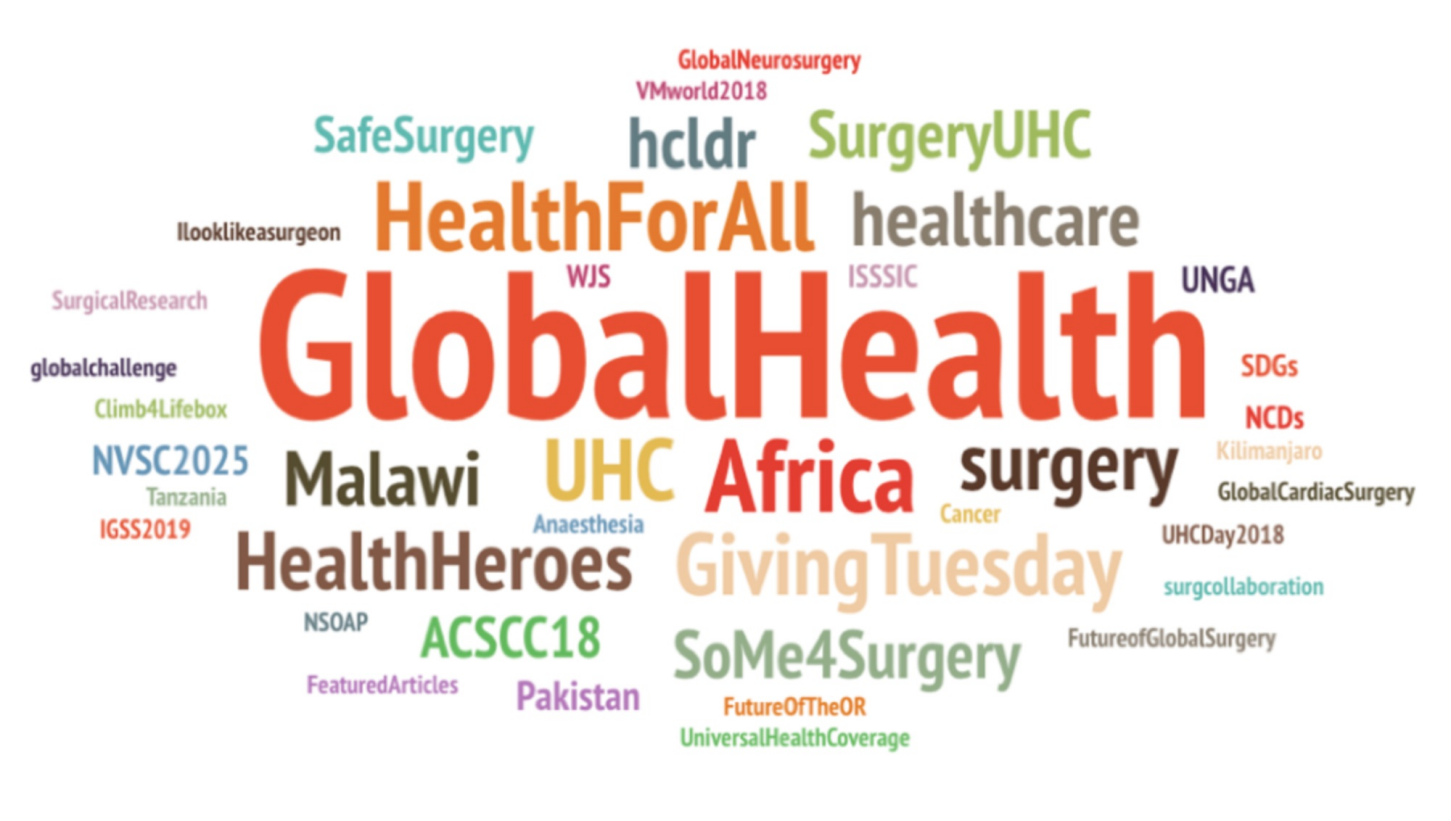
Hashtag cloud. Hashtag cloud of other hashtags included in tweets related to #globalsurgery, with size correlating to the number of impressions made by the relevant tweets.

The global surgery social media community, with communication amongst participants, via the #globalsurgery Hashtag is shown in Figure 2 with a node-edge diagram. Hashtag cloud of other hashtags included in tweets related to #globalsurgery is shown in Figure 3.

We then examined the Twitter activity and followership of a general global surgery Twitter account (Global Alliance for Surgical, Obstetric, Trauma, & Anesthesia Care, The G4 Alliance, @theG4Alliance) as compared to a student-initiated and run global surgery Twitter account (International Student Surgical Network, InciSioN, @InciSioNGlobal, formerly @StudentSurgNet) to a leading global health membership organization Twitter account (Global Health Council, @GlobalHealthOrg). This analysis was done with the purpose to examine the social media impact of these various key players in the #globalsurgery realm.

**Figure 4:**
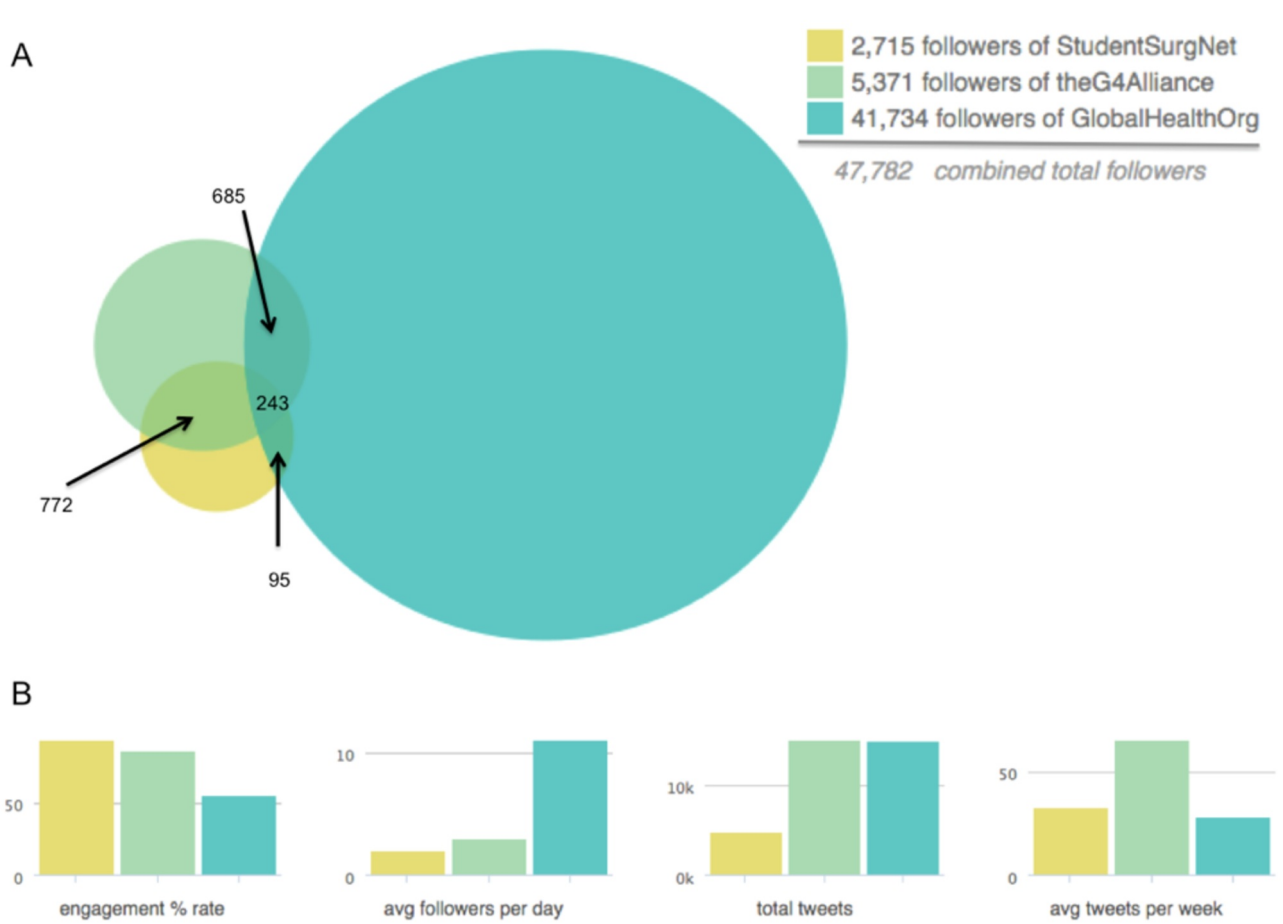
Twitter followership of key stakeholders in #GlobalSurgery advocacy. Twitter followership of InciSioN (@StudentSurgNet, now @InciSioNGlobal), The G4 Alliance (@theG4Alliance), and Global Health Council (GHC) (@GlobalHealthOrg) Twitter accounts with (A) overlap amongst followership and (B) engagement rate (%), average followers per day, total tweets, and average tweets per week.

In January 2019, the Twitter accounts of @StudentSurgNet (@InciSioNGlobal) had a total of 2,715 followers, @theG4Alliance had a total of 5,371 followers, and @GlobalHealthOrg had a total of 41,734 followers, with a respective overlap of 0.5% of follower for all three accounts (Figure 4A). The engagement rates of tweets from the global surgery communities remained high compared to the global health community (@StudentSurgNet 92%, @theG4Alliance 85%, @GlobalHealthOrg 55%); however, they had a lower accumulation of followers per day (@StudentSurgNet 2, @theG4Alliance 3, @GlobalHealthOrg 11) (Figure 4B).

**Figure 5:**
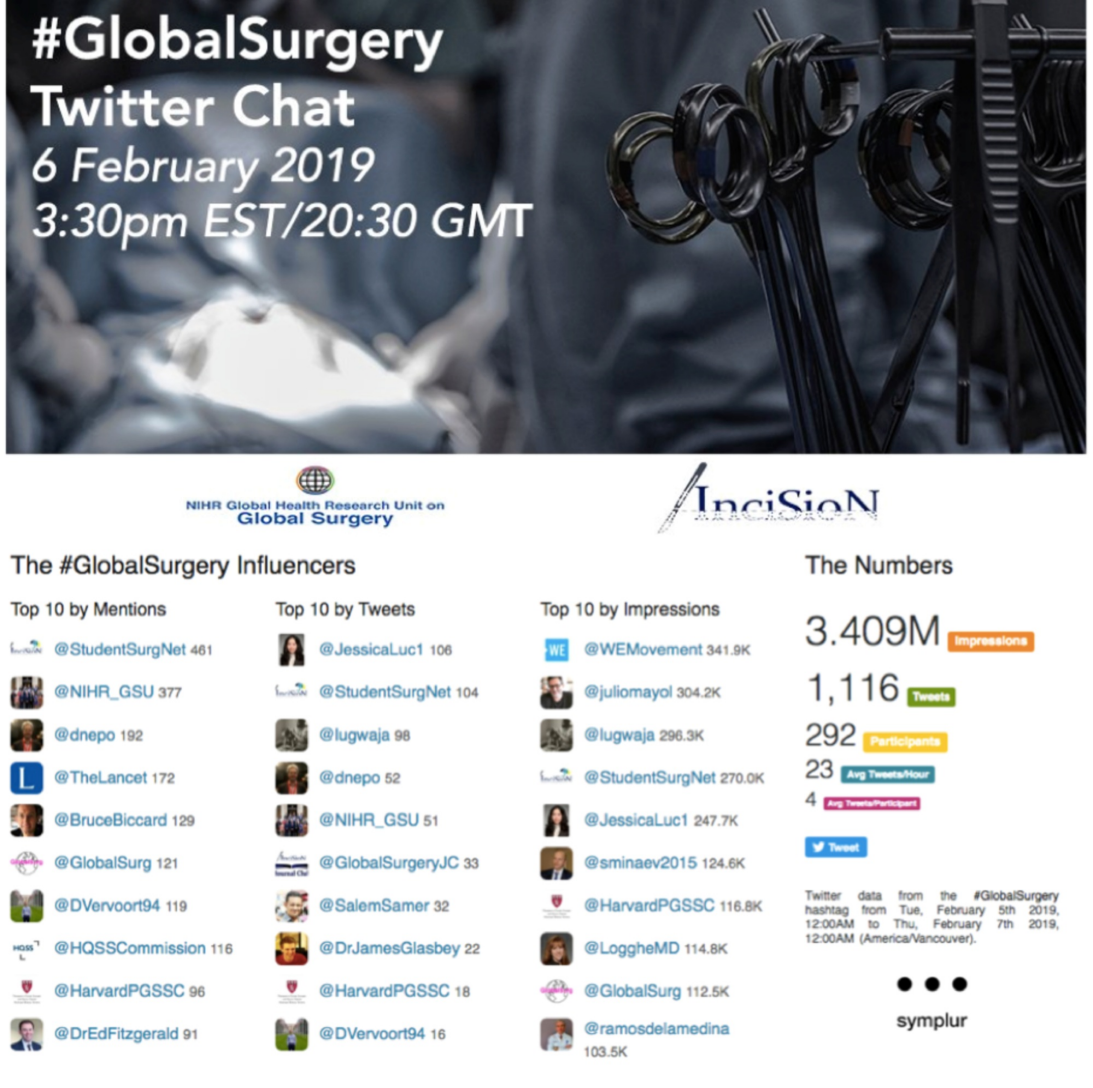
#GlobalSurgery tweetchat analytics obtained from the Symplur online hashtag analytics tool.

A #GlobalSurgery one-hour tweetchat was held by InciSioN (@InciSioNGlobal) in February 2019, drawing a total of 3.4 million impressions, 1,116 tweets, and 292 participants (Figure 5). Global participation of tweetchats attests that they not only served to raise awareness of #GlobalSurgery, but also to strengthen collaboration with the ability to have an international open discussion on the issues and challenges in global surgery and to come up with sustainable solutions. The forum allows both people interested in learning more about the field and seasoned professionals in the field to connect and work towards building a better tomorrow through communication, education, and advocacy.

## Discussion

This study identified the large group of Twitter advocates for global surgery, having engaged in using the hashtag #globalsurgery or a translated hashtag in four of the top five non-English Twitter languages (Japanese, Spanish, Malay, and Portuguese) to form a digitally connected community. In particular, #globalsurgery spanned 4,449 members, 4,495 tweets, and 15,798 retweets with an average impact of 58,714,799 impressions in a period of five months, between July 31, 2018 and December 31, 2018. Tweets were largely positive, encouraging sustained use of positive word markers in #globalsurgery tweets. The Twitter account activity of the analyzed global surgery Twitter accounts had higher engagement rates of tweets per account than a leading global health account; however, they had a lower accumulation of followers per day.

Although the majority (99.47%) of tweets were published in English, people tweeted on global surgery in other languages, such as Japanese and Spanish, as well as Portuguese and Malay when tracing further back in history. However, Malay, the fourth most common language used on Twitter, did not have any usage of #pembedahanglobal in history. There have only been limited tweets including “pembedahan global” after the appointment of Dr. Noor Hisham Abdullah, Director-General of the Ministry of Health in Malaysia, as Chair of the Global Surgery committee in the International Society of Surgery (ISS/SIC) in August 2017. These data create an avenue for increased inclusion of non-English speakers through the translation of tweets and hashtags in other languages.

Surgical organizations play a particularly important role in global surgery on social media, and can have large impact on surgical policies worldwide through its members and social media presence [19]. For example, surgical colleges have a strong following on social media, led by the American College of Surgeons (@AmCollSurgeons; 45,113 followers), followed by the Royal College of Surgeons of England (@RCSnews; 39,579 followers), the Royal College of Surgeons of Edinburgh (@RCSEd; 16,666 followers), and the Royal College of Surgeons in Ireland (@RCSI_Irl; 15,189 followers). Despite their individual contributions to the field of global surgery through capacity-building programs, North-South partnerships, and surgical missions, the colleges have the potential to increase their #globalsurgery efforts, highlighting their own work and bringing the matter to the attention of more people on social media. To illustrate, since the launch of the Lancet Commission on Global Surgery in 2015, @AmCollSurgeons (66 posts), RCSnews (12 posts), and @RCSI_Irl (10 posts), and @RCSEd (3 posts) have not commonly used #globalsurgery in their original tweets. Similarly, encouraging surgical organizations to voice their messages in multiple languages, in particular in local languages that fall within their regional coverage, can target a larger audience that can, in turn, advocate in those languages.

The feasibility analysis demonstrated that tweetchats were an important way to engage the community in open dialogue regarding the issues in global surgery. Tweetchats would be an important tool to continue to host and amplify the discussion and awareness of global surgery; to strengthen collaboration; and to improve communication, education, advocacy, and ultimately clinical practice.

Social media has become an important resource for healthcare professionals in a variety of settings. The advantages include asynchronous and immediate communication across borders and timezones, dynamic online discussions, freely accessible educational content, and instant global reach in a non-discriminatory and low-threshold manner. Twitter has the potential to not only raise awareness of the disparities in access for global surgery but also come up with innovative solutions. Tips on using social media effectively in surgical practice and best practices for surgeons’ social media use have been recently published by the American College of Surgeons [20,21]. There are potential problems with the use of social media, with no formal governing structure, with authors who may or may not be an expert on the subject or therapy discussing issues of global surgery in a global forum with limitless potential, reach, and permanence. However, conversely, allowing everyone to have a seat at the table and have their voices heard is a powerful way that social media democratizes access, ensures diversity and inclusion. it also allows for all positions to be represented. Furthermore, social media is emerging as a form of peer review for experts to call out and correct inaccurate information that may be posted publicly [12].

The study has some limitations. First, it was not possible to obtain historical data due to financial barriers, preventing the ability to assess the growth of the #globalsurgery campaign since the hashtag was first used in 2010. Although no clear trend was observed in the number of tweets, users, or impressions, a trend may have been visible over an extended period of time. Similarly, a retrospective analysis to include the use of #globalsurgery during May 2018 was not possible. This was a month in which high social media activity should have been observed due to Global Surgery Day (25 May), the World Health Assembly, and the InciSioN Global Surgery Symposium 2018; however, our current analysis instead depicts baseline #globalsurgery activity that is not associated with such major events. Secondly, due to monetary reasons and the large volume of tweets, no analysis could be done of the use of the word combination “global surgery”, as opposed to #globalsurgery, in tweets, which could depict a far larger amount of global surgery tweets. Thirdly, misspelled hashtags (e.g., #globalsurgey) were not captured within our analysis; however, manual screening of the use of such variations in tweets during the specified study period indicated that a comparatively negligible number of tweets included misspelled hashtags. Lastly, although the most common Twitter languages other than English were found to have a limited number of global surgery tweets, tweets in other languages are not captured in this analysis. As a result, this contributes to the underestimate of the true global surgery discussions taking place on Twitter.

## Conclusions

With unrestricted access to educational and research opportunities, #globalsurgery provides an innovative method to overcome barriers and strengthen collaboration among surgeons around the world, and spread awareness about global surgery. Surgical specialists and other advocates are empowered to communicate and raise their voices in unison and rapidly on a global scale. Through increased awareness about the cause and escalation of messages through wider social media involvement, policymakers and international leaders can increasingly be held accountable for existing disparities in the health and wealth of nations through fragile and budding surgical systems.
